# Interactive contribution of hyperinsulinemia, hyperglycemia, and mammalian target of rapamycin signaling to valvular interstitial cell differentiation and matrix remodeling

**DOI:** 10.3389/fcvm.2022.942430

**Published:** 2022-10-31

**Authors:** Jessica I. Selig, H. Viviana Krug, Caroline Küppers, D. Margriet Ouwens, Felix A. Kraft, Elena Adler, Sebastian J. Bauer, Artur Lichtenberg, Payam Akhyari, Mareike Barth

**Affiliations:** ^1^Department of Cardiac Surgery, Medical Faculty, University Hospital Düsseldorf, Heinrich Heine University Düsseldorf, Düsseldorf, Germany; ^2^Institute of Clinical Biochemistry and Pathobiochemistry, German Diabetes Center (DDZ), Düsseldorf, Germany; ^3^German Center for Diabetes Research (DZD), Munich, Germany; ^4^Department of Endocrinology, Ghent University Hospital, Ghent, Belgium

**Keywords:** valvular interstitial cells (VIC), calcific aortic valve disease (CAVD), rapamycin, insulin resistance, hyperinsulinemia, hyperglycemia, mammalian target of rapamycin (mTOR), MTORC1/2

## Abstract

Diabetes and its major key determinants insulin resistance and hyperglycemia are known risk factors for calcific aortic valve disease (CAVD). The processes leading to molecular and structural alterations of the aortic valve are yet not fully understood. In previous studies, we could show that valvular interstitial cells (VIC) display canonical elements of classical insulin signaling and develop insulin resistance upon hyperinsulinemia and hyperglycemia accompanied by impaired glucose metabolism. Analyses of cultured VIC and aortic valve tissue revealed extracellular matrix remodeling and degenerative processes. Since PI3K signaling through mammalian target of rapamycin (mTOR) is involved in fibrotic processes of the heart, we aim at further functional investigation of this particular Akt-downstream signaling pathway in the context of diabetes-induced CAVD. Primary cultures of VIC were treated with hyperinsulinemia and hyperglycemia. Phosphorylation of mTOR(Ser^2448^) was determined by Western blot analysis after acute insulin stimulus. Inhibition of mTOR phosphorylation was performed by rapamycin. Phosphorylation of mTOR complex 1 (MTORC1) downstream substrates 4E-BP1(Thr^37/46^) and P70S6K(Thr^389^), and MTORC2 downstream substrate Akt(Ser^473^) as well as the PDK1-dependent phosphorylation of Akt(Thr^308^) was investigated. Markers for extracellular matrix remodeling, cell differentiation and degenerative changes were analyzed by Western blot analysis, semi-quantitative real-time PCR and colorimetric assays. Hyperinsulinemia and hyperglycemia lead to alterations of VIC activation, differentiation and matrix remodeling as well as to an abrogation of mTOR phosphorylation. Inhibition of mTOR signaling by rapamycin leads to a general downregulation of matrix molecules, but to an upregulation of α-smooth muscle actin expression and alkaline phosphatase activity. Comparison of expression patterns upon diabetic conditions and rapamycin treatment reveal a possible regulation of particular matrix components and key degeneration markers by MTORC1 downstream signaling. The present findings broaden the understanding of mitogenic signaling pathways in VIC triggered by hyperinsulinemia and hyperglycemia, supporting the quest for developing strategies of prevention and tailored treatment of CAVD in diabetic patients.

## Introduction

Early disturbances in insulin sensitivity and hyperglycemia (HG) (1, 2) are risk factors for the development of diabetes (3), which is a global burden with increasing prevalence (4, 5). Diabetes in turn is associated with an elevated risk for cardiovascular diseases, including calcific aortic valve disease (CAVD) (6–9). Here, studies report that aortic valve leaflets of diabetics are more prone to inflammation (10), oxidative stress (11) and remodeling (12), and show an enhanced micro-calcification together with an up-regulation of pro-osteogenic markers (13).

In previous work, we could show that valvular interstitial cells (VIC) are sensitive to short-term treatment by diabetic conditions such as hyperinsulinemia (HI) and HG (14). Here, it could be shown that VIC express the insulin receptor, insulin-like growth factor 1 receptor and glucose transporter 1. Beside impaired glucose metabolism, VIC develop an insulin resistance upon HI and HG with disturbed Akt/GSK-3α/β signaling, whilst HG alone was sufficient to induce insulin resistance. Consecutively, alterations in VIC differentiation and early signs of remodeling could be observed (14). Further analysis of aortic valve tissue in a three-dimensional approach revealed that these processes are further modulated by their biomechanical environment and that particular mitogenic signaling pathways such as downstream PI3K signaling are involved (15). Nevertheless, it remains incompletely understood whether HI, HG or the combination of both trigger molecular alterations of VIC and whether downstream PI3K signaling or alternative insulin-sensitive pathways are responsible for these processes.

PI3K signaling through mammalian target of rapamycin (mTOR) is known to be involved in fibrotic processes of several organs and tissues including the myocardium (16). Here, especially the treatment of myocardial dysfunction with mTOR inhibitors like rapamycin has gained attention, particularly in diabetes-related pathologies [reviewed in (17)]. Downstream mTOR signaling is regulated through two mTOR containing complexes: mTOR complex 1 (MTORC1) and MTORC2, both of which have diverse downstream effects and mechanisms in the heart (18). MTORC1 consists amongst others of the adaptor proteins RAPTOR (regulatory-associated protein of mTOR) and DEPTOR (DEP domain containing mTOR-interacting protein). MTORC1 downstream signaling leads to phosphorylation of P70S6K (ribosomal protein S6 kinase 1) and 4E-BP1 (eukaryotic translation initiation factor 4E (eIF4E)-binding protein 1). MTORC2 comprises RICTOR (rapamycin-insensitive companion of mTOR) instead of RAPTOR and activates Akt signaling (17). MTORC1 and MTORC2 signaling have been investigated in the context of myocardial dysfunction and type 2 diabetes (17, 18), whereas knowledge about their role in CAVD and diabetes is still scarce. Understanding of signaling pathways involved in pathological diabetes-induced processes of aortic valve disease, however, is crucial for the development of future preventive or therapeutic strategies. Thus, the present work aims at the investigation of mTOR and mTOR downstream signaling and its role in CAVD.

## Materials and methods

### Primary ovine valvular interstitial cells and treatments

Primary ovine VIC were isolated from aortic valves of fresh ovine hearts (*n* = 7) derived from a local abattoir as described before (14). Aortic valve leaflets were dissected and minced into small pieces. Tissue pieces were incubated in either normoglycemic (NG; 1 g/L glucose) or HG (4.5 g/L glucose) medium in gelatin-covered flasks until VIC grew out. After three to four passages VIC were seeded in gelatin-covered 6-well plates and treated with NG or HG medium with or without 100 nM insulin (Sigma-Aldrich, St. Louis, MO, USA; cat. no. I5523) for 5 days with medium changes every second day [for details please refer to (14)]. Inhibition of mTOR phosphorylation was performed by chronic treatment with 10 nM rapamycin (cat. no. 9904, Cell Signaling, Dallas, TX, USA) using dimethyl sulfoxide (DMSO; Sigma-Aldrich; cat. no. D8418) as vehicle.

For the acute insulin stimulus, supernatant of the cells of all treatment groups was aspirated and the cells were washed twice with PBS. After a starvation period of 4 h in medium without FCS and insulin, VIC were treated with insulin (group: with acute insulin stimulus) or not (group: without acute insulin stimulus). To evaluate the optimal insulin concentration cells were initially treated either with 10 nM or with 100 nM insulin for 10 min. In the further process, 10 nM insulin was used for the acute stimulus. Afterward, cells were washed twice with cold PBS and were lysed for SDS-PAGE and Western blot as described before (14).

### SDS-PAGE and Western blot analysis

Lysates were conducted to SDS-PAGE and separated proteins were blotted on nitrocellulose. Protein signals were detected using the following primary antibodies purchased from Cell Signaling: mTOR (cat. no. 2983); phospho-mTOR(Ser^2448^) (cat. no. 2971); 5′-adenosine monophosphate (AMP)-activated protein kinase (AMPKα; cat. no. 2603); phospho-AMPKα(Thr^172^) (cat. no. 2535); ribosomal protein S6 kinase beta-1 (P70S6K; cat. no. 2708); phospho-P70S6K(Thr^389^) (cat. no. 9234); 4E-BP1 (cat. no. 9452); phospho-4E-BP1(Thr^37/46^) (cat. no. 9459); Akt (cat. no. 4691), phospho-Akt(Ser^473^) (cat. no. 4060), and phospho-Akt(Thr^308^) (cat. no. 13038). Detection of housekeeping protein GAPDH (cat. no. 2118) or β-actin (cat. no. 4967) on the according membranes was used for normalization of protein signals. Signals of primary antibodies were detected by using the following secondary antibodies: Goat IgG anti-rabbit IgG (H + L)-HRP (cat. no. 111-035-003, Jackson ImmunoResearch, Ely, UK) and goat IgG anti-mouse IgG & IgM (H + L)-HRP (cat. no. 115-035-044, Jackson ImmunoResearch). Molecular weight was determined by using a PageRuler Prestained Protein ladder (cat. no. 26616; Thermo Fisher Scientific, Waltham, MA, USA). Detection of protein signals was performed by using an Amersham Imager 600 (GE Healthcare, Chalfont St Giles, UK) and intensity of protein bands was analyzed by Image Quant TL software (GE Healthcare). Detection of 4E-BP1 and Akt as well as the corresponding phosphorylated antibodies was performed with IRDye 800 CW goat anti rabbit (cat. no. 926-32211, LI-COR biosciences, Lincoln, NE, USA) and IRDye 680 LT goat anti mouse (cat. no. 926-68020, LI-COR biosciences) as secondary antibodies using an Odyssey scanner (LI-COR biosciences).

Western blot images of Supplementary Figure 1 and Figures 3, 4 were cropped since additional conditions have been tested for control experiments on these blots. Uncropped images of the appropriate blots are depicted in Supplementary Figures 2–4, respectively.

### mRNA analysis by semi-quantitative real-time PCR

Isolation of total RNA, cDNA synthesis as well as semi-quantitative real-time PCR was performed as previously described (10). Primer sequences were as follows: collagen type 1 (*COL1A1*; forward 5′-AAGACATCCCACCAGTCACC-3′, reverse 5′-TAAGTTCGTCGCAGATCACG-3′), elastin (*ELN*; forward 5′- AGTTCCTGGAGGCGTCTTCT-3′, reverse 5′ CAC CTGGCTTAGCTGGTTTC-3′), biglycan (*BGN*; forward 5′-TCTGCTCCGCTACTCCAAGT-3′, reverse 5′-TTGTTGTCC AAGTGCAGCTC-3′), decorin (*DCN*; forward 5′ CCAAAGTG CGAAAGTCTGTG-3′, reverse 5′-TTCAATGCCTGAGCTCT TCA-3′), α-smooth muscle actin (*ACTA2*; forward 5′-GATA GAGCACGGCATCATCA-3′, reverse 5′-GAAGGGTTGGATG CTCTTCA-3′), osteopontin (*OPN*; forward 5′-GATGGCCG AGGTGATAGTGT-3′, reverse 5′-TCGTCTTCTTAGGTGCG TCA-3′), alkaline phosphatase (*ALP*; forward 5′-CAACACC AACGTGGCTAAGA-3′, reverse 5′-GTTGTGGTGGAGCTG ACCTT-3′), matrix metalloproteinase 2 (*MMP2*; forward 5′-TGACAAGGACGGCAAGTATG-3′, reverse 5′-GTAAGATGT GCCCTGGAAGC-3′), hyaluronic acid synthase 2 (*HAS2*; forward 5′-TCACCCAGTTGGTCTTGTCC-3′, reverse 5′-GG TCAAGCATGGTGTCTGAA-3′), and matrix metalloprotei nase 9 (*MMP9*; forward 5′-TAGCACGCACGACATCTTTC-3′, reverse 5′-GCCCACATAGTCCACCTGAT-3′). Relative mRNA expression analysis was performed by 2^–ΔΔ*Ct*^ method using CT means of the following reference genes: β-tubulin (forward 5′-CCTACAACTGGACCGCATCT-3′, reverse 5′-AAAGGACCTGAGCGAACAGA-3′), 60S ribosomal protein L13a (*RPL13a*; forward 5′-GATCCCACCACCCTATGACA-3′, reverse 5′-CTTCAGACGCACAACCTTGA-3′), and 60S ribosomal protein L29 (*RPL29*; forward 5′-CCAAGTCCAAG AACCACACC-3′, reverse 5′-TATCGTTGTGATCGGGGTTT-3′). In the following, gene names are written in italics.

### Detection of alkaline phosphatase activity

Using the PierceTM PNPP Substrate Kit (cat. no. 37620, Thermo Fisher Scientific), the concentration of cell released alkaline phosphatase (*n* = 3) was measured using supernatants of the media before inhibitor treatment and then every second day. The assay was performed according to the manufacturers’ instructions using a microplate reader (Infinite M1000 Pro) by measuring the absorbance at 405 nm.

### Detection of cytotoxicity

The CyQUANT™ LDH cytotoxicity assay (cat. no. C20300, Thermo Fisher Scientific) was used to determine the amount of lactate dehydrogenase (LDH) released into the media of the cells. Therefore, supernatants were collected at the day of cell harvest and the colorimetric assay was performed according to the manufacturer’s instructions using a microplate reader (Infinite M1000 Pro). Data was normalized to total protein content.

### Statistical analysis

Statistical analysis was performed using GraphPad Prism version 7 (GraphPad Software, San Diego, CA, USA). Data is presented as mean ± SEM. Statistical analysis of data originating from Western blot analysis was performed using two-way ANOVA with Sidak’s multiple comparison test. Statistical analysis of data originating from semi-quantitative real-time PCR, ALP and LDH analysis was performed using Kruskal–Wallis test with Dunn’s *post hoc* test. For comparison of two groups, Mann–Whitney U test or Wilcoxon signed rank test was applied. *p*-Values < 0.05 were considered as statistically significant.

## Results

### Hyperglycemia and hyperinsulinemia abrogate PI3K signaling in valvular interstitial cells

**FIGURE 1:**
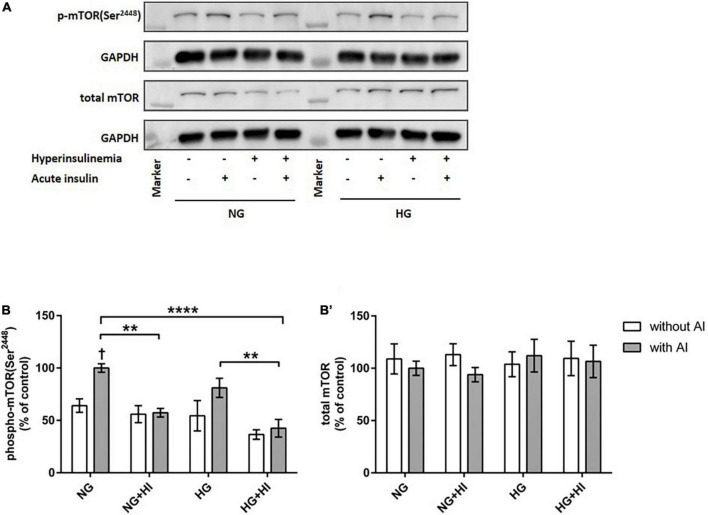
Diabetic conditions abrogate mTOR phosphorylation. Phosphorylation of mTOR(Ser^2448^) upon hyperinsulinemia under normoglycemia and hyperglycemia was measured in cultured ovine VIC (*n* = 7). Representative Western blot images show phosphorylated and total mTOR expression **(A)**. Quantification of protein signals showed that hyperinsulinemia led to significantly decreased phosphorylation levels of mTOR(Ser^2448^) as well as to an abrogation of inducible phosphorylation upon acute insulin stimulus **(B)**. Total amount of mTOR was not altered **(B’)**. Data was normalized to GAPDH and expressed relative to normoglycemic conditions with acute insulin stimulus. NG, normoglycemia; HI, hyperinsulinemia; HG, hyperglycemia; AI, acute insulin stimulus. **p-Values < 0.01 and ****p-values < 0.0001 between indicated groups; ^†^*p*-values < 0.05 compared to basal condition without acute insulin stimulus. Lanes of protein ladder represent 35 and 250 kDa, respectively.

**FIGURE 2:**
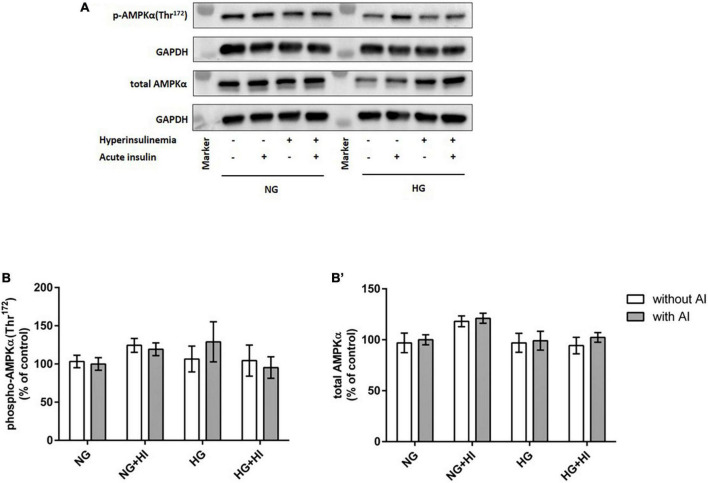
AMPK phosphorylation is not impaired by diabetic conditions. Phosphorylation of AMPK(Thr^172^) upon hyperinsulinemia under normoglycemia and hyperglycemia was measured in cultured ovine VIC (*n* = 7). Representative Western blot images show phosphorylated and total AMPKα expression **(A)**. Quantification of protein signals showed that neither acute insulin stimulus nor hyperinsulinemia or hyperglycemia led to alterations in AMPK(Thr^172^) phosphorylation **(B)**. Total amount of AMPK was not altered by the treatments **(B′)**. Data was normalized to GAPDH and expressed relative to normoglycemic conditions with acute insulin stimulus. NG, normoglycemia; HI, hyperinsulinemia; HG, hyperglycemia; AI, acute insulin stimulus. Lanes of protein ladder represent 35 and 70 kDa, respectively.

To elucidate the impact of diabetic conditions on PI3K signaling, Western blot analysis of mTOR signaling was performed in VIC (Figure 1). Acute insulin stimulation led to a significant increase in mTOR(Ser^2448^) phosphorylation (*p* = 0.012) under NG, an effect which was abrogated by treatment with HI or HG as well as by the combination of both treatments. Upon acute insulin stimulus, HI inhibited phosphorylation of mTOR(Ser^2448^) under NG treatment by 43% (*p* = 0.003) as well as under HG treatment by 48% (*p* = 0.009) (Figures 1A,B). Compared to NG conditions, the combination of HG and HI led to a significant decrease of mTOR(Ser^2448^) phosphorylation upon acute insulin stimulus by 57% (*p* < 0.0001). HG treatment alone did not lead to a significant decrease of mTOR(Ser^2448^) phosphorylation upon acute insulin stimulus compared to the appropriate NG condition. Basal phosphorylation (Figures 1A,B) as well as protein abundance of total mTOR (Figure 1B’) was not affected by diabetic conditions. In contrast to mTOR, AMPK signaling was not impaired by diabetic conditions (Figure 2).

### Impact of diabetic conditions and mammalian target of rapamycin inhibition on mammalian target of rapamycin complex 1 and mammalian target of rapamycin complex 2 signaling

For rapamycin treatment, the optimal rapamycin concentration was evaluated, revealing that 10 nM rapamycin efficiently decreased basal phosphorylation of mTOR(Ser^2448^) (Supplementary Figure 1) whilst cell morphology was not affected (not shown). In order to evaluate mTOR signaling, downstream targets of MTORC1 and MTORC2 were investigated.

#### Mammalian target of rapamycin complex 1 signaling upon diabetic conditions and rapamycin treatment

**FIGURE 3:**
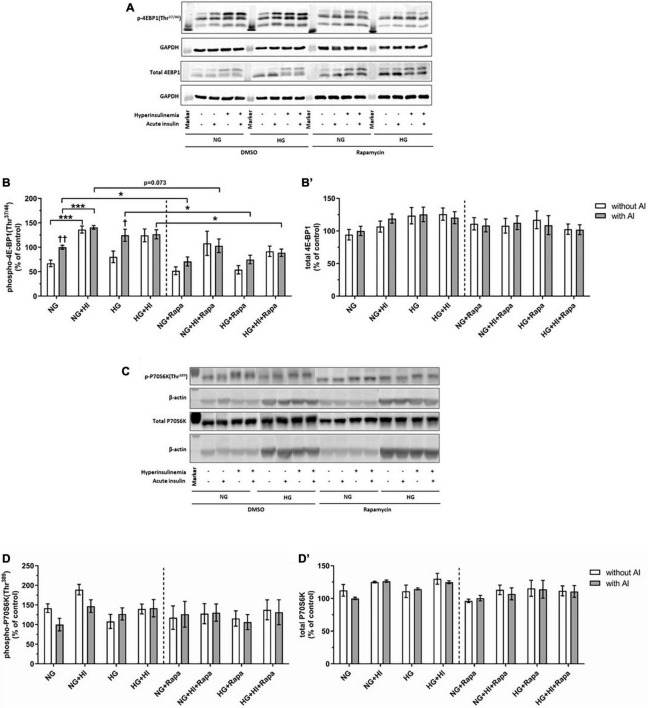
Mammalian target of rapamycin complex 1 signaling upon diabetic conditions and rapamycin treatment. Phosphorylation of 4E-BP1(Thr^37/46^) and P70S6K(Thr^389^) upon hyperinsulinemia under normoglycemia and hyperglycemia was measured in cultured ovine VIC (*n* = 6 for 4E-BP1 and *n* = 4 for P70S6K). Representative Western blot images show phosphorylated and total 4E-BP1 **(A)** and P70S6K expression **(C)**. Quantification of 4E-BP1 protein signals showed that rapamycin treatment led to generally lower phosphorylation levels of 4E-BP1(Thr^37/46^) upon acute insulin stimulus. In absence of rapamycin, acute insulin stimulus led to significantly higher phosphorylation levels of 4E-BP1(Thr^37/46^) whilst hyperinsulinemia abolished the effect of inducible phosphorylation **(B)**. Total amount of 4E-BP1 was not altered **(B′)**. Quantification of P70S6K protein signals showed that neither acute insulin stimulus nor hyperinsulinemia or hyperglycemia led to alterations in P70S6K(Thr^389^) phosphorylation **(D)**. Total amount of P70S6K was not altered by the treatments **(D′)**. Data was normalized to GAPDH or β-actin, and expressed relative to normoglycemic conditions with acute insulin stimulus. NG, normoglycemia; HI, hyperinsulinemia; HG, hyperglycemia; AI, acute insulin stimulus; Rapa, rapamycin. *p-Values < 0.05 and ***p-values < 0.001 between indicated groups; ^†^*p*-values < 0.05 and ^†⁣†^*p*-values < 0.01 compared to basal condition without acute insulin stimulus. Lanes of protein ladder represent 15 kDa (4E-BP blots) and 35 kDa (GAPDH blots) as well as 70 kDa (P70S6K blots) and 35 kDa (β-actin blots), respectively.

Hyperinsulinemia under NG conditions led to a significantly higher 4E-BP1(Thr^37/46^) phosphorylation both under basal conditions (*p* = 0.0006) and upon acute insulin stimulus (*p* = 0.0006). Acute insulin stimulus led to a significant increase of 4E-BP1(Thr^37/46^) under NG (*p* = 0.007) as well as under HG (*p* = 0.038) conditions compared to the corresponding basal conditions, whereas HI treatment abrogated this effect. Rapamycin treatment led to attenuated inducible 4E-BP1(Thr^37/46^) phosphorylation by acute insulin (Figures 3A,B). Generally, upon acute insulin stimulus, rapamycin treatment led to a lower phosphorylation levels of 4E-BP1(Thr^37/46^) both under NG (*p* = 0.038) and NG + HI (*p* = 0.073) conditions as well as under HG (*p* = 0.018) and HG + HI (*p* = 0.014) conditions compared to the corresponding untreated group (Figures 3A,B). Basal phosphorylation as well as total 4E-BP1 abundance was not altered (Figure 3B’). Phosphorylation of P70S6K(Thr^389^) was not altered by rapamycin treatment (Figures 3C,D). Total P70S6K was not altered (Figure 3D’).

#### Mammalian target of rapamycin complex 2 signaling upon diabetic conditions and rapamycin treatment

**FIGURE 4:**
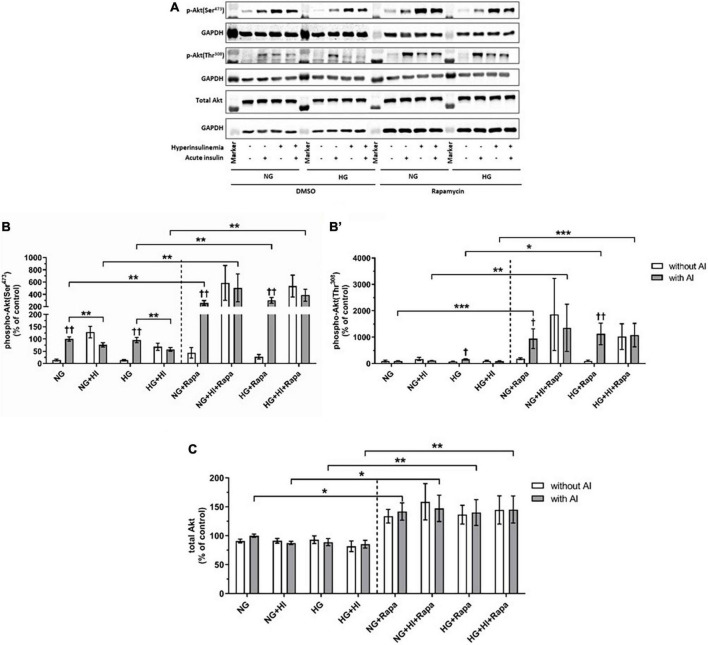
Mammalian target of rapamycin complex 2 signaling upon diabetic conditions and rapamycin treatment. Phosphorylation of Akt(Ser^473^) and Akt(Thr^308^) upon hyperinsulinemia under normoglycemia and hyperglycemia was measured in cultured ovine VIC (*n* = 6). Representative Western blot images show phosphorylated and total Akt expression **(A)**. Quantification of Akt protein signals showed that rapamycin treatment led to a generally higher Akt(Ser^473^) **(B)** and Akt(Thr^308^) **(B′)** phosphorylation upon acute insulin stimulus. Acute insulin stimulus led to significantly higher phosphorylation levels of Akt(Ser^473^) whilst hyperinsulinemia abolished the effect of inducible phosphorylation under control conditions as well as upon rapamycin treatment **(B)**. Akt(Thr^308^) phosphorylation was significantly upregulated by acute insulin stimulus under HG conditions **(B′)**. Upon rapamycin treatment, hyperinsulinemia abolished inducible phosphorylation Akt(Thr^308^) by acute insulin stimulus **(B′)**. Total amount of Akt showed a generally higher expression level upon rapamycin treatment, whereas neither acute insulin stimulus nor hyperinsulinemia or hyperglycemia led to alterations of Akt expression **(C)**. Data was normalized to GAPDH and expressed relative to normoglycemic conditions with acute insulin stimulus. NG, normoglycemia; HI, hyperinsulinemia; HG, hyperglycemia; AI, acute insulin stimulus; Rapa, rapamycin. *p-Values < 0.05, **p-values < 0.01, and ***p-values < 0.001 between indicated groups; ^†^*p*-values < 0.05 and ^†⁣†^*p*-values < 0.01 compared to basal condition without acute insulin stimulus. Lanes of protein ladder represent 70 and 55 kDa (Akt blots) and 35 kDa (GAPDH blots), respectively.

Compared to the corresponding basal group, acute insulin stimulus led to a significantly increased phosphorylation of Akt(Ser^473^) under NG (*p* = 0.002) and HG (*p* = 0.002) treatment (Figures 4A,B). HI treatment abrogated inducible phosphorylation of Akt(Ser^473^) and led to a significantly reduced Akt(Ser^473^) phosphorylation under NG + HI (*p* = 0.0022) as well as under HG + HI treatment (*p* = 0.0026) upon acute insulin stimulus compared to NG and HG alone. A similar effect was observed upon rapamycin treatment; here, phosphorylation was increased under NG (*p* = 0.004) and HG (*p* = 0.002) treatment upon acute insulin stimulus compared to basal conditions and was abrogated by HI treatment. Generally, treatment with rapamycin led to a significantly increased phosphorylation level of Akt(Ser^473^) upon acute insulin stimulus under NG (*p* = 0.002), NG + HI (*p* = 0.004), HG (*p* = 0.002) and HG + HI (*p* = 0.002) conditions (Figures 4A,B) compared to the according untreated group.

Detection of Akt(Thr^308^) phosphorylation proved to be difficult due to generally low expression levels and unspecific binding (Figure 4B’). Acute insulin stimulus led to an increased phosphorylation under HG conditions in the untreated group (*p* = 0.011), whereas phosphorylation was not inducible under NG (*p* = 0.620), NG + HI (*p* = 0.805) and HG + HI (*p* = 0.710) conditions. Under rapamycin treatment, acute insulin stimulus led to an increased Akt(Thr^308^) phosphorylation compared to the according basal condition under NG (*p* = 0.018) as well as under HG (*p* = 0.007) conditions. HI treatment abrogated this effect (Figure 4B’). Similar to phosphorylation of Akt(Ser^473^), rapamycin treatment led to generally higher expression levels of phosphorylated Akt(Thr^308^) upon acute insulin stimulus compared to the according untreated groups (NG: *p* = 0.0006; NG + HI: *p* = 0.005; HG: *p* = 0.018; HG + HI: *p* = 0.0006). Total Akt abundance was not influenced by acute insulin stimulus compared to basal conditions. However, rapamycin led to generally higher Akt expression levels compared to the untreated groups (Figure 4C).

### Valvular interstitial cells differentiation and matrix remodeling under diabetic conditions and mammalian target of rapamycin inhibition

Next, we analyzed the impact of diabetic conditions as well as the impact of rapamycin as an inhibitor of mTOR signaling on VIC differentiation and matrix remodeling. For rapamycin treatment, the optimal rapamycin concentration was evaluated, revealing that 10 nM rapamycin efficiently decreased basal phosphorylation of mTOR(Ser^2448^) (Supplementary Figure 1) whilst cell morphology was not affected (not shown).

#### Valvular interstitial cells activation and viability

Upon stress or tissue damage, VIC change their quiescent fibroblastoidal phenotype toward an activated myofibroblastoidal phenotype with higher contractility, which is indicated by an increased expression of ACTA2 (19–21). The term “VIC activation” used in this work thus refers to this phenomenon, and does not necessarily involve concomitant enhanced matrix remodeling as commonly described for pathological changes in CAVD (recently reviewed in (22).

**FIGURE 5:**
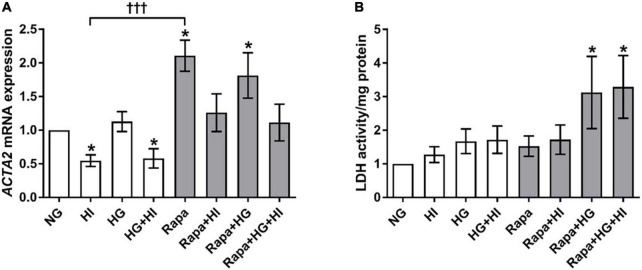
Valvular interstitial cells activation and viability under diabetic conditions and mTOR inhibition. Impact of diabetic conditions and mTOR inhibition on gene expression of *ACTA2* **(A)** and on LDH activity **(B)** was analyzed. HI led to a decrease of *ACTA2* gene expression, whereas rapamycin led to a remarkable and statistically highly significant opposite effect. *ACTA2*, α-smooth muscle actin; LDH, lactate dehydrogenase; HI, hyperinsulinemia; HG, hyperglycemia; Rapa, rapamycin. *p-Values < 0.05 of direct comparison to normoglycemic control; ^†⁣†⁣†^*p*-value < 0.001 of intergroup comparisons.

Hyperinsulinemia treatment led to a significant downregulation of *ACTA2* gene expression in VIC under NG (*p* = 0.016) as well as under HG conditions (*p* = 0.047) compared to NG control conditions. HG treatment alone had no effect (*p* = 0.469). Inhibition of mTOR signaling by rapamycin treatment led to a significant upregulation of *ACTA2* gene expression under NG (*p* = 0.016) as well as under HG conditions (*p* = 0.031) compared to NG control conditions. HI treatment seemed to mitigate these effects. Comparison between rapamycin and HI treatment revealed opposed effects with highly significant difference (*p* = 0.0005; Figure 5A). LDH activity as a marker for cytotoxicity was not significantly altered by diabetic conditions, nor by rapamycin treatment under NG conditions. Rapamycin treatment in combination with HG treatment, however, led to a significantly higher LDH activity both without HI (*p* = 0.047) and with HI (*p* = 0.047) when compared to NG conditions (Figure 5B).

#### Structural extracellular matrix molecules and matrix metalloproteinases

**FIGURE 6:**
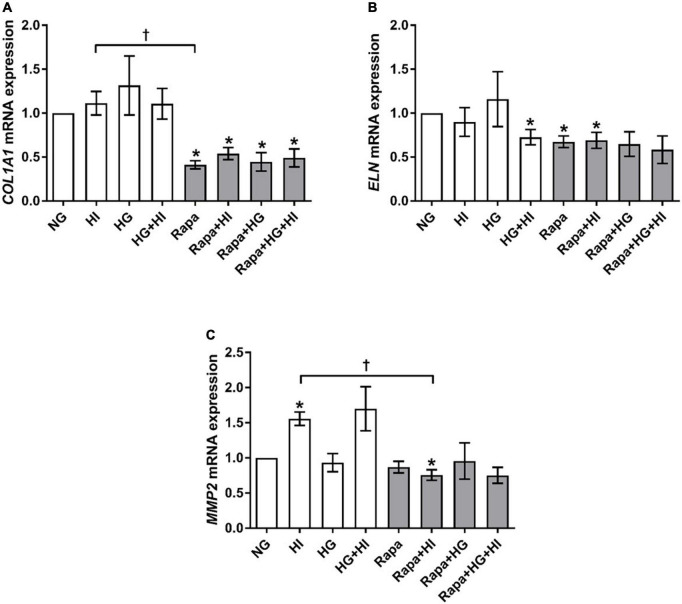
Expression of structural extracellular matrix molecules and matrix metalloproteinase under diabetic conditions and mTOR inhibition. Impact of diabetic conditions and mTOR inhibition on gene expression of *COL1A1* **(A)**, *ELN* **(B)**, and *MMP2* **(C)** was analyzed. Diabetic conditions had no significant impact on *COL1A1* gene expression, whereas rapamycin treatment led to a reduction of *COL1A1* gene expression **(A)**. *ELN* gene expression was reduced by HG + HI as well as by rapamycin treatment compared to NG conditions **(B)**. *MMP2* gene expression was induced by HI. Rapamycin + HI led to decreased *MMP2* gene expression. *COL1A1*, collagen type 1; *MMP2*, matrix metalloproteinase 2; *ELN*, elastin; HI, hyperinsulinemia; HG, hyperglycemia; Rapa, rapamycin. *p-Values < 0.05 of direct comparison to normoglycemic control; ^†^*p*-values < 0.05 of intergroup comparisons.

*COL1A1* gene expression was not significantly altered by diabetic conditions. Rapamycin treatment in contrast, led to significantly decreased *COL1A1* gene expression compared to NG control conditions independent of diabetic conditions (*p* = 0.016; Figure 6A). Comparison between rapamycin and HI treatment revealed significantly lower *COL1A1* gene expression under rapamycin treatment (*p* = 0.033). Gene expression of *ELN* was significantly downregulated by HG + HI treatment with similar effect upon rapamycin treatment alone (*p* = 0.016) as well as in combination with HI (*p* = 0.031) when compared to NG control conditions (Figure 6B). Combined treatment of rapamycin with HG or HG + HI conditions led to a slight trend toward lower *ELN* gene expression in comparison to NG control conditions (*p* = 0.078). *MMP2* gene expression was significantly upregulated under HI conditions (*p* = 0.016) and by a slight trend under HG + HI conditions (*p* = 0.078; Figure 6C). Comparison between HI and rapamycin treatment revealed a visible difference, however not statistically different (*p* = 0.323), whereas comparison between HI and HI + rapamycin showed a significant opposed effect (*p* = 0.018). *MMP9* gene expression levels were not detectable (not shown).

#### Glycosylated matrix molecules

**FIGURE 7:**
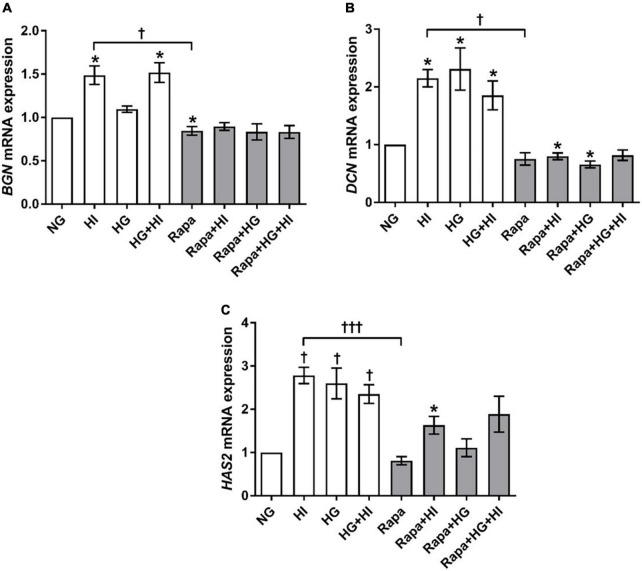
Expression of glycosylated matrix molecules under diabetic conditions and mTOR inhibition. Impact of diabetic conditions and mTOR inhibition on *BGN* **(A)**, *DCN* **(B)**, and *HAS2* **(C)** gene expression was analyzed. *BGN* gene expression is upregulated by HI but downregulated by rapamycin treatment **(A)**. *DCN* gene expression was upregulated by diabetic conditions, whereas rapamycin treatment did not alter *DCN* gene expression **(B)**. *HAS2* gene expression was upregulated by diabetic condition. Rapamycin treatment alone did not alter *HAS2* gene expression, whereas combination with HI led to increased expression levels **(C)**. *BGN*, biglycan; *DCN*, decorin; *HAS2*, hyaluronic acid synthase 2; HI, hyperinsulinemia; HG, hyperglycemia; Rapa, rapamycin. *p-Values < 0.05 of direct comparison to normoglycemic control; ^†^*p*-values < 0.05 of intergroup comparisons in relation to NG treatment; ^†⁣†⁣†^*p*-values < 0.001 of intergroup comparisons.

Analysis of glycosylated matrix molecules revealed a significant upregulation of *BGN* gene expression upon HI treatment (*p* = 0.031) as well as upon HG + HI treatment (*p* = 0.016; Figure 7A). Rapamycin treatment led to a numerically small effect but significantly lower *BGN* gene expression when compared to NG control conditions (*p* = 0.031). Comparison between HI and rapamycin treatment showed significantly different gene expression of *BGN* (*p* = 0.010). Interestingly, when the subset of rapamycin-containing treatments is considered, rapamycin entirely abolished the effects of diabetic conditions on the expression of *BGN* as observed within the subset of conditions without rapamycin. *DCN* gene expression was in general upregulated by diabetic conditions when compared to NG control conditions (*p* = 0.016; Figure 7B). Rapamycin treatment in combination with HI or HG conditions led to numerically small but significantly lower *DCN* gene expression when compared to NG control conditions (*p* = 0.016). Comparison between HI and rapamycin treatment showed significantly different gene expression of *DCN* (*p* = 0.008). Similar to the observations on *BGN* gene expression, rapamycin led to a general abrogation of the elevating effect of diabetic conditions on *DCN* gene expression. *HAS2* gene expression was significantly upregulated by diabetic conditions either by HI (*p* = 0.003), HG (*p* = 0.019) or combined treatment with HI + HG (*p* = 0.037; Figure 7C). Rapamycin treatment in contrast, did not alter *HAS2* gene expression, whereas additional stimulation with HI led to significantly higher *HAS2* gene expression when compared to NG control conditions (*p* = 0.016). Comparison between HI and rapamycin treatment showed significantly different *HAS2* gene expression levels (*p* = 0.0004), with an overall inhibition of the stimulatory impact of diabetic conditions on *HAS2* gene expression.

#### Valvular interstitial cells chondro-osteogenic differentiation

**FIGURE 8:**
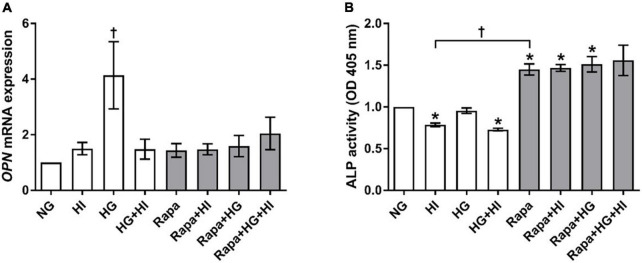
Expression of degeneration markers under diabetic conditions and mTOR inhibition. Impact of diabetic conditions and mTOR inhibition on *OPN* gene expression **(A)** and ALP activity **(B)** was analyzed. HG treatment led to a significant upregulation of *OPN* gene expression, whereas HI as well as rapamycin treatment kept *OPN* gene expression low **(A)**. ALP activity was reduced by HI treatment, whereas rapamycin treatment led to increased ALP activity. *OPN*, osteopontin; ALP, alkaline phosphatase; HI, hyperinsulinemia; HG, hyperglycemia; Rapa, rapamycin. *p-Values < 0.05 of direct comparison to normoglycemic control; ^†^*p*-values < 0.05 of intergroup comparisons as indicated or in relation to NG treatment.

*OPN* gene expression was significantly upregulated by HG treatment (*p* = 0.023). Rapamycin treatment did not alter *OPN* gene expression. However, *OPN* gene expression levels upon HI or HI + HG treatment were comparable to those under rapamycin treatment (Figure 8A). HI and HG + HI treatment led to significantly decreased ALP activity when compared to NG control conditions (*p* = 0.031; Figure 8B). Rapamycin treatment, however, led to a generally higher ALP activity when compared to NG control conditions (*p* = 0.031) except for the combination with HG + HI which led to a trend toward higher ALP activity (*p* = 0.063). Comparison between HI and rapamycin treatment revealed significantly opposed effects on ALP activity (*p* = 0.027). Collectively, rapamycin abrogated the inducing effect of HG on *OPN* gene expression and had a strong enhancing effect on ALP activity that contrasts the effects observed under HI.

## Discussion

The present work shows that chronic insulin treatment leads to decreased mTOR signaling by abrogating mTOR phosphorylation in VIC. Chronic insulin exposure as well as HG conditions in turn lead to VIC activation, chondro-osteogenic differentiation, and matrix remodeling. Inhibition of mTOR signaling by rapamycin altered the impact of diabetic conditions with respect to various components of the extracellular matrix as well as of markers of activation and chondro-osteogenic differentiation. Remarkably, the effect of mTOR inhibition in front of effects observed for diabetic conditions was not uniform: Rapamycin treatment resulted in a modulation of VIC regulation, partly resembling an amplification of HI or HG induced effects whilst also reflecting a marked inhibitory impact on other factors. These results suggest that mTOR is involved in intracellular transmission of diabetic stimulus on VIC but is not the only signaling pathway by which diabetic conditions regulate aortic valve morphology.

### Mammalian target of rapamycin complex 1 and mammalian target of rapamycin complex 2 signaling under diabetic conditions

Hyperinsulinemia led to a decreased susceptibility of 4E-BP1(Thr^37/46^) for phosphorylation upon acute insulin stimulus. This might be indicative for an impaired MTORC1 downstream signaling in VIC upon chronic diabetic conditions, as it has been described before for HUVECS and smooth muscle cells *in vitro*. Here, impaired phosphorylation of 4E-BP1 has been observed even after 5 min of HI (23). However, skeletal muscle of diabetics did not show alterations in 4E-BP1 phosphorylation (24). In contrast to reports on renal epithelial cells showing increased 4E-BP1 phosphorylation under HG treatment (25), this was not the case for VIC under HG treatment. Response to diabetic conditions thus seems to differ between different cell types and tissues and the presented results suggest that VIC share an alternating subset of features with several other cell types. Similar to 4E-BP1(Thr^37/46^), a decrease of P70S6K(Thr^389^) phosphorylation has been described *in vitro* in HUVECS and smooth muscle cells after a short-time incubation with HI (23). Reports focused on cancer cells showed an increase in phosphorylation levels of P70S6K after 15 min of HI followed by an adaptation to long-term exposure to HI (26). In the present work, phosphorylation levels of P70S6K(Thr^389^) did not show significant changes under diabetic conditions in VIC which might be related to specific differences in short-term vs. long-term HI treatments. Time course experiments as well as examination of valvular tissue of diabetics might therefore lead to a better understanding of diabetes-induced P70S6K(Thr^389^) phosphorylation in VIC. However, it has been shown that P70S6K acts *via* different phosphorylation sites by MTORC1 activation alone or in combination with other kinases (27, 28). Thus, future experiments might also aim at the investigation of alternative phosphorylation sites of P70S6K besides (Thr^389^).

As we have shown before, in the present work HI led to impaired Akt(Ser^473^) signaling under NG as well as under HG conditions (14). Under the applied conditions, Akt(Thr^308^) phosphorylation is not as sensitive to HI as Akt(Ser^473^) phosphorylation. Moreover, Akt(Thr^308^) phosphorylation seems to be mainly impaired by HG conditions. Susceptibility of both Akt(Ser^473^) and Akt(Thr^308^) phosphorylation to impaired signaling upon HI is more pronounced under rapamycin treatment. Here, Akt(Thr^308^) phosphorylation shows a similar pattern as Akt(Ser^473^) phosphorylation. This effect here seen in VIC might probably be due to MTORC1 inhibition by rapamycin and a resulting inhibition of the negative feedback loop, which then enhances activation of Akt(Ser^473^) signaling (29) thereby triggering subsequent Akt(Thr^308^) signaling. However, enhanced susceptibility of Akt(Thr^308^) phosphorylation by prior Akt(Ser^473^) activation has been shown already by Sarbassov et al. (30).

### Mammalian target of rapamycin complex 1 and mammalian target of rapamycin complex 2 signaling under mammalian target of rapamycin inhibition

Rapamycin was initially considered as a potent inhibitor of translation and thus, e.g., inhibiting tumor growth through inhibition of MTORC1 complex and downstream inhibition of 4E-BP1 and P70S6K signaling (31). However, inhibition of 4E-BP1 phosphorylation of, e.g., 4E-BP1(Thr^37/46^) by rapamycin seems to be unstable in long-term treatment (48 h +) and is particularly described to be cell type dependent (32). However, the effect of rapamycin on MTORC1 downstream signaling and especially on 4E-BP1 and P70S6K phosphorylation has scarcely been described for VIC (33). Our data show that rapamycin is able to decrease 4E-BP1(Thr^37/46^) phosphorylation in VIC upon acute insulin stimulus even after 5 days of treatment. In addition, rapamycin also reduces the susceptibility of 4E-BP1 to inducible phosphorylation by acute insulin stimulus.

Moreover, phosphorylation of P70S6K(Thr^389^) was not influenced by rapamycin treatment in VIC. This might be due to the relatively low rapamycin concentration chosen for our experiments (10 nM), whereas others also reported cell line dependent responsiveness with this concentration (34). Murine VIC treated with 100 nM rapamycin showed a decrease in P70S6K phosphorylation (35), which might be indicative for the need of higher concentrations of rapamycin. Apart from the mentioned report based upon a 1-h treatment with rapamycin (35), to our knowledge, long-term effects on P70S6K have not yet been reported for VIC. However, adverse effects of rapamycin or time-dependent suspension of mTOR pathway inhibition has been reported before for other cells (32). Effects of *in vivo* long-term rapamycin treatment on MTORC1 downstream targets P70S6K and 4E-BP1 have been described recently for vascular tissue showing a reduction of these both MTORC1 targets (36). Interestingly, inhibition of the phosphorylation of these two MTORC1 downstream targets by rapamycin is described to be temporarily different. In the reported model, inhibition of P70S6K phosphorylation alleviates over time, whereas inhibition of 4E-BP1 phosphorylation aggravates after day 7 after administration (36). Evaluation of a time- and/or dose-dependent rapamycin action or the impact or more specific mTOR inhibitors like torin on the responsiveness of VIC therefore might be necessary to investigate the role of 4E-BP1 and P70S6K in this setting in detail.

Previous reports have suggested that short-term rapamycin treatment inhibits MTORC1 whereas long-term treatment inhibits also MTORC2 and impairs subsequent Akt signaling (37). The definition of “long-term” treatment though is ambiguous since clinical application certainly is not comparable to *in vitro* experimental treatments. However, impact of rapamycin treatment on MTORC2 has been described to be cell type dependent (37) and dependent on the time of rapamycin treatment or administration, respectively. Reports on administration of rapamycin *in vivo* up to 21 days describe unchanged MTORC2 downstream activation in vascular tissue (36) whereas *in vitro* treatment of fibroblasts with rapamycin for 4 days abolished Akt signaling (38).

Based on this, it is not clear whether MTORC2 is impaired by 5 days treatment with rapamycin in our approach, since we do not see impaired Akt signaling but rather increased Akt phosphorylation of both Akt(Ser^473^) und Akt(Thr^308^). Since Akt(Thr^308^) is not mTOR dependent, we did not expect a decrease here, but would have excepted an impaired Akt(Ser^473^) phosphorylation. Increase in Akt signaling in VIC might therefore be due to a not yet sufficient inhibition of MTORC2 together with the inhibition of the negative feedback loop of MTORC1 leading to enhanced activation of Akt signaling (29, 39). As Sarbassov et al. have reported earlier, also PI3K/PDK1 regulated Akt(Thr^308^) phosphorylation depends on Akt(Ser^473^) phosphorylation (30) and can be influenced by rapamycin treatment, which has been also shown recently in CRISPR/Cas9-based knockout of RICTOR (40). Nevertheless, our data on Akt signaling cannot dissect whether a possible inactivation of MTORC2 took place and if so, to which extend this would influence Akt(Ser^473^) phosphorylation when compared to the enhanced activation due to a loss of MTORC1 negative feedback. In conclusion, interplay of MTORC1/MTORC2 in the balance of Akt phosphorylation is delicate and cell type specific, even more so with respect to the question of the impact of long-term usage of rapamycin.

### Impact of diabetic conditions on valvular interstitial cells differentiation and valvular matrix

Diabetes is associated with a higher risk to develop aortic stenosis (7, 9) however, knowledge about the impact of HI and HG on aortic valve molecular composition is still limited. Aortic valves of diabetics are more prone to chondro-osteogenic differentiation and matrix remodeling than the valves of non-diabetics (11- 13). Animal studies analyzing the pathophysiology of the aortic valve in presence of diabetes further support the findings of clinical observations (41, 42). The present work shows that HI and HG as hallmarks of diabetes significantly alter the aortic valve on a cellular level, i.e., VIC differentiation and matrix remodeling. Similar to previous investigations, HI suppresses activation of VIC (14), whereas HG alone did not alter *ACTA2* expression, which was also observed by others (43). Diabetic conditions generally increased matrix remodeling, indicated by elevated expression of *MMP2* and proteoglycans, mainly but not exclusively due to HI treatment. HG treatment alone altered *DCN* and *HAS2* gene expression but was not sufficient to alter other matrix molecules as has been reported for VIC in three-dimensional *in vitro* approaches (43). Here, biomechanical stimuli seem to enhance some effects on matrix remodeling, an observation which we could corroborate in previous studies involving aortic valve tissue culture in different biomechanical environments (15).

It is surprising that ALP activity in VIC is rather downregulated by HI, since aortic valve tissue of human diabetics (13) as well as aortic valve tissue in a murine diabetes-induced atherosclerosis model (42) showed increased levels of ALP. Discrepancies in these findings may be due to the isolated investigation of VIC with selected stimuli, i.e., HG and HI, *versus* the complex pathophysiology involving dyslipidemia and inflammation, both of which have been described to take place in diabetes. Taken together, HI and HG treatment lead to molecular alterations of VIC, which can be also found in preclinical models of diabetes-induced aortic valve degeneration.

### Impact of rapamycin on valvular interstitial cells differentiation and valvular matrix

Mammalian target of rapamycin signaling mediates fibrotic remodeling of several tissues including the myocardium (16). Inhibition of mTOR by rapamycin or so-called “rapalogs” is used to prevent heart failure and cardiac remodeling [reviewed in (18, 44)]. Such pharmacological intervention has also been suggested as a promising approach to prevent myocardial dysfunction in diabetes (45, 46). Nevertheless, reports on the role of mTOR in degenerative changes in cardiac valves, and specifically in VIC are scarce (47). In the present work, rapamycin treatment of VIC evokes a general downregulation of gene expression of several matrix components like *COL1A1*, *ELN* as well as of *BGN* and *DCN*. However, effects of mTOR inhibitors vary amongst cell types of different origin and with diverse actions of rapalogs of different generations or depending on the applied techniques (e.g., RNA interference vs. Rapalink-1), providing heterogeneous or even contrary findings, e.g., for proteoglycans (48, 49) or matrix metalloproteinases (50, 51). In the present work, rapamycin treatment led to an increased gene expression of *ACTA2*. Interestingly, especially the effect on alpha smooth muscle actin (α-SMA) activation seems to be cell type dependent as well as susceptible to different mTOR inhibitors. For example, an increase in α-SMA expression was reported for vascular smooth muscle cells (52) and mesangial cells upon sirolimus treatment (53). In contrast, an inhibitory effect of rapamycin on α-SMA expression could be shown in endothelial-like cells (38) and fibroblasts (39). Long-term treatment with rapamycin led to a reduced α-SMA expression together with impaired MTORC2 downstream signaling *via* Akt in fibroblasts (38). A lack of decreased *ACTA2* expression in our approach thus might imply that our 5 days rapamycin treatment has yet not been enough to provoke inhibition of MTORC2 signaling with subsequent inhibition of myofibroblastoidal differentiation of VIC. However, these effects seem to be highly dependent on the starving status of the cells, since opposite effects have been described for unstarved cells (38).

Osteogenic markers such as osteopontin or alkaline phosphatase in contrast, seem to be in general rather downregulated by rapamycin as it has been shown for VIC (48) as well as for vascular smooth muscle cells (52). Discrepancies between our findings of unchanged *OPN* gene expression and an increased ALP activity and reports on downregulation of these markers might therefore be due to different treatment durations, an issue that is also discussed in the clinical application of mTOR inhibitors (54). Taken together, in our setting, rapamycin treatment leads to an activation of VIC together with an upregulation of ALP activity. Thus, the role of ALP may be subjected to further translational studies and might be a promising candidate for clinical investigation.

### Potential involvement of mammalian target of rapamycin signaling in valvular interstitial cells differentiation and matrix remodeling under diabetic conditions

Our analyses show that chronic insulin treatment leads to an abrogation of mTOR phosphorylation in VIC even after short-time cultivation. In contrast to findings on upstream Akt signaling (14), HG conditions alone are not sufficient to impair mTOR signaling. Here, the reduction of mTOR phosphorylation was highest under combined HG conditions and chronic insulin exposure. Furthermore, AMPK is characterized as an upstream mTOR inhibitor, sensing glucose changes and inhibiting mTOR in episodes of glucose starvation (55, 56). In the present work, AMPK phosphorylation was not altered by varying glucose concentrations, which might be indicative for a mainly aerobic metabolism of VIC (57). Moreover, previous studies have shown that VIC mainly express the insulin-independent glucose transporter 1 but not glucose transporter 4 (14), which would be regulated by AMPK in case of metabolic imbalance (58).

In front of the background that diabetic conditions deplete mTOR signaling, the question arises which mitogenic effect induced by diabetic conditions can be ascribed to a regulation by mTOR signaling. Here, effects of rapamycin treatment should lead to similar expression patterns if not even further pronounced patterns as compared to the treatment with diabetic conditions. This effect was present for the gene expression of *ELN*, a structural matrix component, which was significantly downregulated both by treatment with HI + HG as well as by rapamycin treatment. The same holds true for *OPN* expression as a marker for chondro-osteogenic differentiation of VIC, which is upregulated by HG treatment, whereas HI treatment impedes this effect. Here, rapamycin treatment led to similar expression patterns. This is indicative for diabetes induced matrix remodeling and differentiation being mediated by mTOR signaling at this point. Most of the other investigated targets in the present work did not show comparable patterns of reaction. Concerning certain effects, even contrary reactions between diabetic conditions and rapamycin treatment were observed, e.g., in case of *ACTA2*, *MMP2*, and *BGN* gene expression as well as in case of ALP activity. In the case of latter subjects, the alterations induced by diabetic conditions do not seem to be mediated by mTOR signaling but rather by alternative pathways such as sonic hedgehog signaling, which has been described as relevant for osteoblastic differentiation under HG (59).

Concerning the question whether these observations can be attributed to mTOR downstream signaling *via* MTORC1 or MTORC2, similar effects in VIC provoked by diabetic conditions and rapamycin treatment might be seen in MTORC1 downstream 4E-BP1 signaling, where HI impairs susceptibility of VIC to induced phosphorylation, which can be also shown for rapamycin. Nevertheless, future investigations are needed to dissect the detailed involvement of MTORC1 downstream signaling in the course of VIC differentiation, remodeling and degeneration under diabetic conditions and mTOR inhibition.

## Conclusion

Diabetic conditions lead to molecular alterations of VIC as well as to impaired mTOR signaling with different susceptibility to HG and HI. Inhibition of mTOR signaling by rapamycin treatment results in decreased matrix expression but also in an activation of VIC together with an upregulation of ALP activity. Particular matrix components and chondro-osteogenic markers reveal an mTOR-mediated reaction of VIC to diabetic conditions with potential involvement of MTORC1 downstream signaling *via* impaired 4E-BP1 phosphorylation. However, these data underscore the need for further investigations using MTORC1-specific inhibitors. Moreover, these findings foster the hypothesis of diabetes-induced initiation and progression of CAVD and further suggest that the involved molecular events may be only partly mediated by mTOR signaling. Further investigations therefore might aim at tailored treatment with mTOR inhibitors considering patients at increased risk for the development of CAVD, i.e., diabetics.

## Limitations of the study

Hyperinsulinemia and HG are hallmarks of diabetic complications. However, isolated application of these two factors does not reflect the pathophysiology of diabetes with its complex aspects of inflammation, disturbed protein metabolism and dyslipidemia. Thus, the findings of the present work focus on the isolated effects of insulin and glucose on mTOR signaling, which should be further validated in translational studies, i.e., animal models of diabetes.

The use of *in vitro* [two-dimensional (2D)] cell culture bears several limitations, e.g., the influence of the interaction with other cell types of the tissue of origin, the lack of cell–matrix interactions or, especially in the case of the aortic valve, the lack of shear stress-induced mechanisms. Thus, findings of *in vitro* experiments have to be regarded with great attention concerning their comparability with whole tissue examinations and need to be confirmed by three-dimensional analysis and in translational approaches. Nevertheless, *in vitro* culture environments offer advantages for basic studies of isolated parameters if used in a reproducible and stable model.

The use of a non-human (i.e., ovine) VIC cell source is also afflicted with limitations, since these cells will differ from the corresponding human source in relation to mechanics, physiology, and immunology. Although ovine VIC have advanced to a reliable model for functional studies on CAVD, direct comparability to human-derived VIC is restricted and validation in human-derived cell lines therefore is inevitable. Our approach using ovine VIC is mainly owed to the lack of human healthy donor valves and a heterogeneous population of patients concerning age, medication and co-morbidities. Ovine VIC therefore represent a “healthy” phenotype that is hardly achievable with human specimen and allows analysis without patient-related side effects.

Although rapamycin is a clinically proven agent for the treatment of heart failure and post-infarction cardiac remodeling, it might lead to conflicting or confounding results *in vitro*, since it is inferior in completely blocking MTORC1 together with MTORC2 in comparison to, e.g., torin (60, 61). Thus, findings concerning rapamycin-induced effects on MTORC1 have to be interpreted carefully.

In the present investigations, DMSO was used as a solvent for rapamycin. DMSO is known to have unwarranted effects on cell proliferation and differentiation, which we sought to minimize by using concentrations of 0.1% DMSO.

## Data availability statement

The original contributions presented in this study are included in the article/Supplementary material, further inquiries can be directed to the corresponding author.

## Author contributions

MB and JS: conceptualization, formal analysis, and project administration. MB, JS, DO, and PA: methodology. JS, MB, and PA: validation. HK, CK, FK, and EA: investigation. AL: resources. HK and MB: data curation and visualization. MB and PA: writing—original draft preparation. MB, JS, AL, DO, SB, and PA: writing—review and editing. JS, MB, AL, and PA: supervision. JS: data curation and visualization. All authors contributed to the article and approved the submitted version.
