# Supplementary material for: Interactive contribution of hyperinsulinemia, hyperglycemia, and mammalian target of rapamycin signaling to valvular interstitial cell differentiation and matrix remodeling
Source: Front Cardiovasc Med. 2022 Oct 31;9:942430. doi: 10.3389/fcvm.2022.942430 (PMC9661395; doi:10.3389/fcvm.2022.942430)
Supplement: Supplementary file 1 [file Data_Sheet_1.PDF]

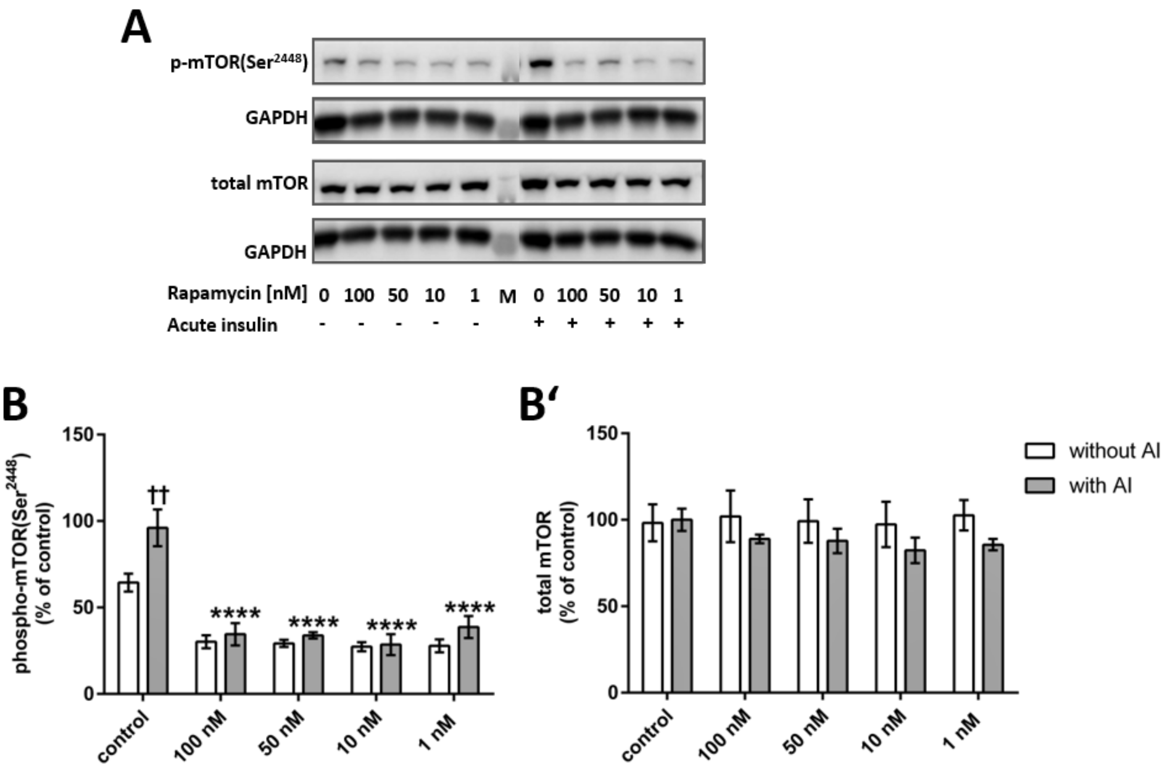

Supplemental Figure 1

Evaluation of optimal concentrations of rapamycin (A-B') was performed in cultured ovine VIC (n=4). Rapamycin concentrations of 1, 10, 50 and 100 nM led to significantly reduced expression of phosphorylated mTOR(Ser2448) under acute insulin simulation (A+B) together with abrogation of induced upregulation of phosphorylated mTOR(Ser2448) upon acute insulin stimulus. Total mTOR expression was not affected (B'). A rapamycin concentration of 10 nM was chosen for further analyses. Data was normalized to GAPDH and expressed relative to normoglycemic control conditions with acute insulin stimulus. NG: normoglycemia; HI: hyperinsulinemia; HG: hyperglycemia; AI: acute insulin stimulus; ****: p-values <0.0001 compared to control condition with acute insulin stimulus; ††: p-values <0.01 compared to basal condition without acute insulin stimulus. Lanes of protein ladder represent 35 kDa and 250 kDa, respectively.
