# Supplementary material for: Interactive contribution of hyperinsulinemia, hyperglycemia, and mammalian target of rapamycin signaling to valvular interstitial cell differentiation and matrix remodeling
Source: Front Cardiovasc Med. 2022 Oct 31;9:942430. doi: 10.3389/fcvm.2022.942430 (PMC9661395; doi:10.3389/fcvm.2022.942430)
Supplement: Supplementary file 2 [file Data_Sheet_2.PDF]

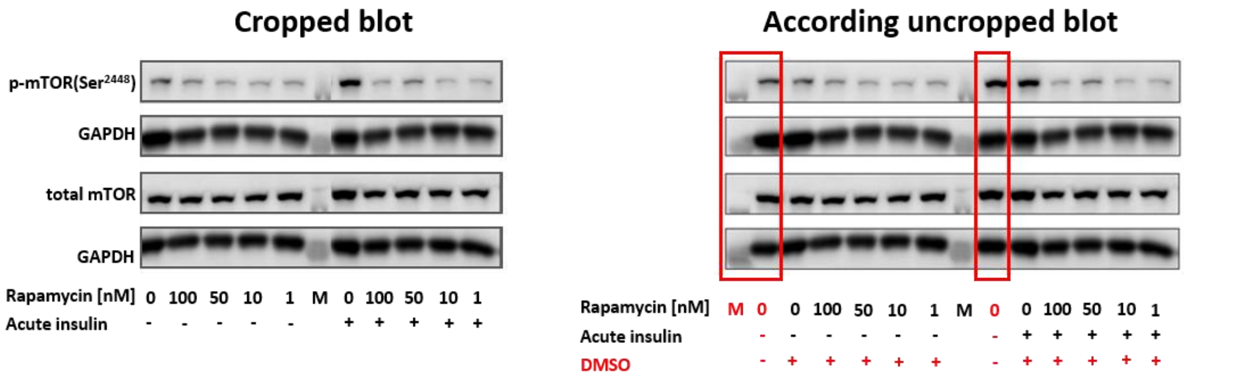

Supplemental Figure 2

Declaration of cropped Western blot image in Supplemental Figure 1 depicting the cropped blot (left) and the according uncropped blot (right). Lanes in red boxes in the right picture have been removed since these were loaded with cell lysates incubated without DMSO and rapamycin to evaluate possible vehicle effects. NG: normoglycemia; HI: hyperinsulinemia; HG: hyperglycemia; AI: acute insulin stimulus; Lanes of protein ladder represent 35 kDa and 250 kDa, respectively.
