# Supplementary material for: Interactive contribution of hyperinsulinemia, hyperglycemia, and mammalian target of rapamycin signaling to valvular interstitial cell differentiation and matrix remodeling
Source: Front Cardiovasc Med. 2022 Oct 31;9:942430. doi: 10.3389/fcvm.2022.942430 (PMC9661395; doi:10.3389/fcvm.2022.942430)
Supplement: Supplementary file 3 [file Data_Sheet_3.PDF]

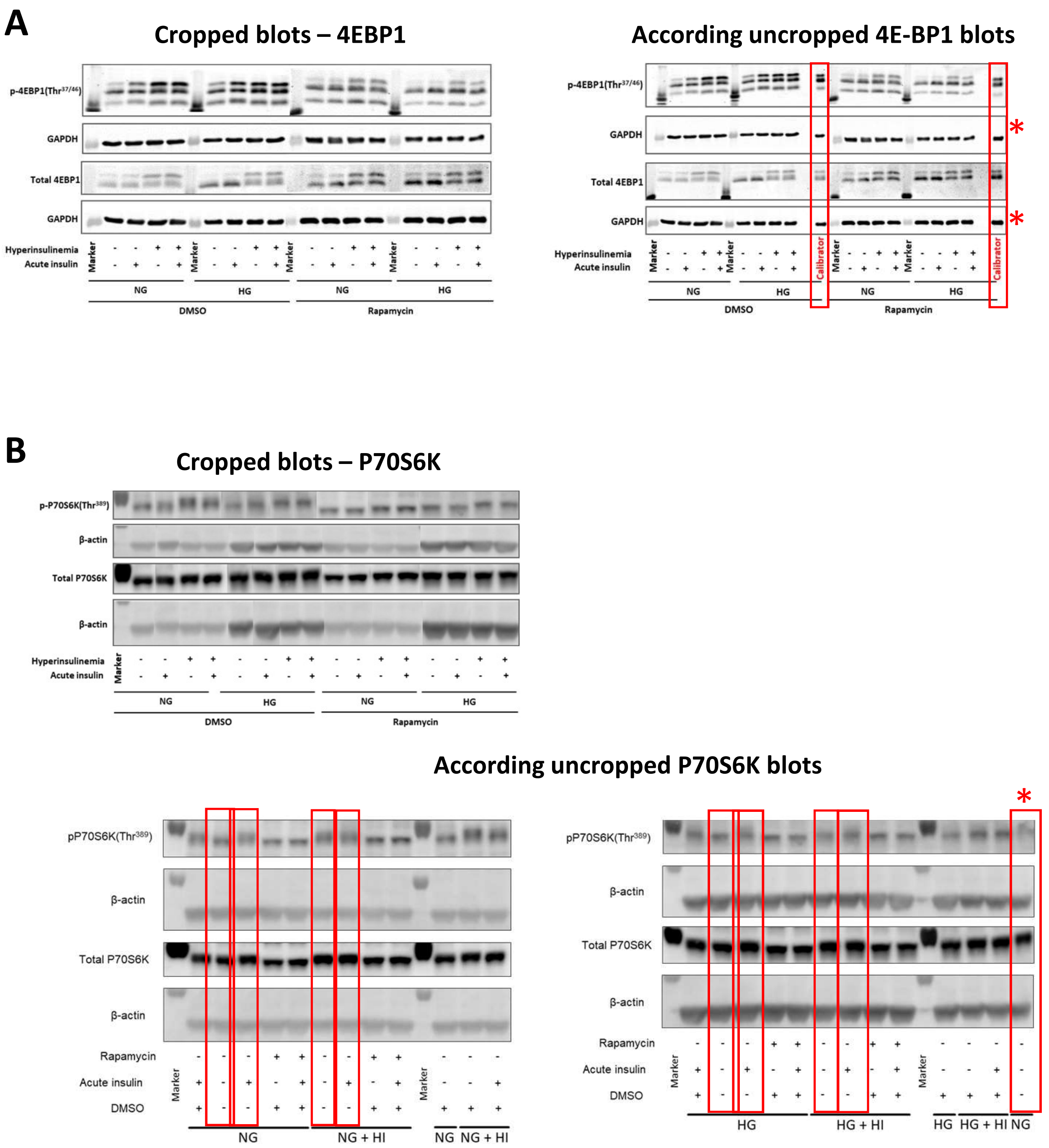

Supplemental Figure 3

(A) Declaration of cropped Western blot image of 4EBP1 in Supplemental Figure 3 depicting the cropped blot (left) and the according uncropped blot (right). Lanes in red boxes in the right picture have been removed since these were loaded with cell used for calibration and comparison of blots run with samples with DMSO and with rapamycin. *: p-Akt(Ser473) and 4E-BP1(Thr37/46) as well as total Akt and 4E-BP1 shared the same membrane, so that GAPDH loading controls sometimes are the same, depending on the replicate chosen for descriptive pictures (please see Supplemental Figure 4).

(B) Declaration of cropped Western blot image of P70S6K in Supplemental Figure 3 depicting the cropped blot (left) and the according uncropped blot (right). Lanes in red boxes in the right picture have been removed since these were loaded with cell lysates incubated without DMSO and rapamycin to evaluate possible vehicle effects. *: Lane in the right picture has been removed since this was loaded with cell lysate used for calibration and comparison of blots run with samples with NG and with HG treatment.

NG: normoglycemia; HI: hyperinsulinemia; HG: hyperglycemia; AI: acute insulin stimulus; Lanes of protein ladder represent 15 kDa (4E-BP blots) and 35 kDa (GAPDH blots) as well as 70 kDa (P70S6K blots) and 35 kDa (β-actin blots), respectively.
