# Supplementary material for: Interactive contribution of hyperinsulinemia, hyperglycemia, and mammalian target of rapamycin signaling to valvular interstitial cell differentiation and matrix remodeling
Source: Front Cardiovasc Med. 2022 Oct 31;9:942430. doi: 10.3389/fcvm.2022.942430 (PMC9661395; doi:10.3389/fcvm.2022.942430)
Supplement: Supplementary file 4 [file Data_Sheet_4.PDF]

Cropped blots – Akt **According uncropped Akt blots**

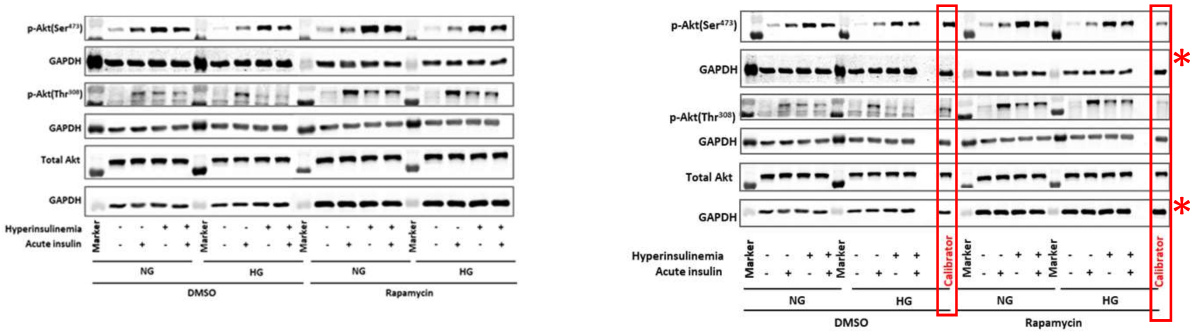

Supplemental Figure 4

Declaration of cropped Western blot image in Supplemental Figure 4 depicting the cropped blot (left) and the according uncropped blot (right). Lanes in red boxes in the right picture have been removed since these were loaded with cell lysates used for calibration and comparison of blots run with samples with DMSO and with rapamycin. NG: normoglycemia; HI: hyperinsulinemia; HG: hyperglycemia; AI: acute insulin stimulus; *: p-Akt(Ser^{473}) and 4E-BP1($Thr^{37/46}$) as well as total Akt and 4E-BP1 shared the same membrane, so that GAPDH loading controls sometimes are the same, depending on the replicate chosen for descriptive pictures (please see Supplemental Figure 3). Lanes of protein ladder represent 70 and 55 kDa (Akt blots) and 35 kDa (GAPDH blots), respectively.
